# Asymptomatic Mediastinal Extra-adrenal Paraganglioma as a Cause of Sudden Death: A Case Report

**DOI:** 10.1515/biol-2019-0062

**Published:** 2019-12-31

**Authors:** Qiancheng Xu, Yingya Cao, Hongzhen Yin, Rongrong Wu, Tao Yu, Weihua Lu

**Affiliations:** 1 Department of Critical Care Medicine, Research Center for functional maintenance and reconstruction of viscera, Wannan Medical College First Affiliated Hospital, Yijishan Hospital, Wuhu, China, 241000; 2 Department of Education, Wannan Medical College First Affiliated Hospital, Yijishan Hospital, Wuhu, China, 241000

**Keywords:** Cardiothoracic surgery, Endocrine

## Abstract

A 23-year-old female patient was referred for treatment of a posterior mediastinal tumour. There was no history of hypertension or headache and no other complaints. The patient’s blood pressure increased to 210/125 mmHg after surgically manipulating the tumour, subsequently reversing to severe hypotension (25/15 mmHg) immediately after the tumour was removed. The life-threatening and irreversible blood pressure drop was difficult to treat with fluid and vasopressors, and the patient ultimately died of cardio-respiratory failure. Asymptomatic paraganglioma can be non-functional but can also be fatal. For any lump in the thoracic cavity, paraganglioma should be ruled out.

## 1 Background

Pheochromocytoma arises from neuro-ectodermal tissue that secretes catecholamines, causing hypertension and headache. Most pheochromocytomas originate from the adrenal gland; nevertheless, they can arise from anywhere along the sympathetic chain among the paraganglion tissues, usually in the abdomen but even in the pelvis, head, neck and thorax, and are termed

paragangliomas. The incidence of pheochromocytomas in the general population is 0.001%–0.01%. Diagnosis of pheochromocytoma is usually made on the basis of clinical presentation and elevated catecholamine levels in serum or urine. Imaging is used to locate the tumours. Asymptomatic pheochromocytoma that is totally clinically silent is difficult to diagnosis pre-operatively. We report a case of asymptomatic mediastinal paraganglioma undiagnosed until surgery, resulting in sudden death.

## 2 Case presentation

**Figure 1:**
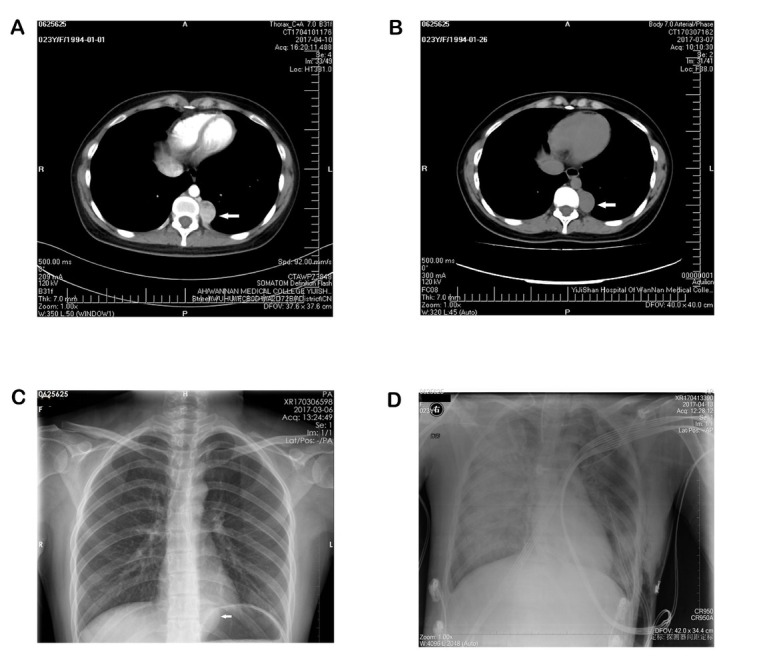
Radiologic examination. (A/B) Thoracic contrast-enhanced CT showing a 2.5x2 cm mass at left posterior mediastinum (white arrow) with heterogeneous density. (C) Posteroanterior chest x-ray showing a left posterior mediastinum soft-tissue mass (white arrow). (D) Chest X-ray 12 hours after operation showing severe interstitial and alveolar oedema.

A 23-year-old female patient was referred for treatment of a posterior mediastinal tumour to the left of the spinal column that was found incidentally on a chest CT during physical examination one month prior (Figure 1 A/B). There was no history of hypertension or headache or any other complaint. She was admitted to the cardiothoracic surgery department for pre-operative preparation. Her vital signs were normal, with blood pressure ranging from 110/60 to 125/74 mmHg. Electrocardiography and echocardiography were normal. Posteroanterior chest x-ray film revealed revealed a left posterior mediastinum soft-tissue mass (white arrow) (Figure 1 C), All laboratory examination results were almost within normal limits. Enhanced computed tomography of the chest revealed a posterior mediastinal mass close to the left paravertebral column that was hypervascularized and well-demarcated, measuring 2.5×2 cm. Based on all of these examinations, the pre-operative diagnosis was a mediastinal mass, probably benign.

**Figure 2:**
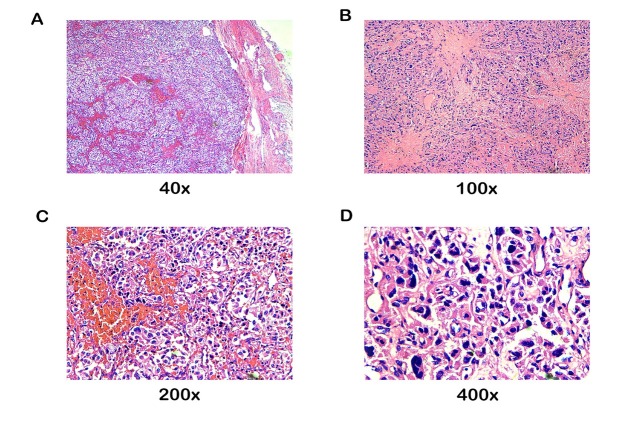
The microscopic findings show characteristic arrangement in well-defined nests (zellballen) bound by a delicate fibrovascular stroma. The tumor cells vary in size and shape and have a finely granular basophilic or amphiphilic cytoplasm with mild cellular atypia.

On the third day, the patient underwent thoracoscopic mediastinal mass resection though the seventh intercostal space in the mid-axillary axial line and the fifth intercostal space in the front axillary axial line, revealing a tumour situated in the left paravertebral sulcus, appearing with a smooth, complete capsule. When we peeled off the capsule surrounding the tumour, the patient’s blood pressure rapidly increased to 210/125 mmHg with a

decrease in oxygen saturation. A paraganglioma was strongly suspected, and the operation was discontinued. Phentolamine and esmolol were used to control blood pressure and heart rate. Surgery was resumed after the vital signs had returned to normal. When the tumour was completely resected, the patient’s blood pressure dropped sharply to 25/15 mmHg. Fluids (colloid and crystalline liquids and albumin) and catecholamines were infused without delay. These interventions did not work: vital signs (HR: 149 bpm, SPO2: 70%, BP: 86/50 mmHg) remained unstable despite high-dose norepinephrine (2.08 μg/kg/min) and adrenaline (0.42 μg/kg/min) and ventilator support (FIO2: 100%, PEEP: 15 cmH2O). Blood gas analysis showed severe hyperlacticaemia and metabolic acidosis (pH 7.341, Lac 11.5 mmol/L, BE -4.1 mmol/L). Laboratory examination showed troponin I (TropI) 29.02 ng/ml, BNP 1342.40 pg/ml, methaemoglobin 2027.4 ng/mL and creatine kinase-MB 18.1 ng/mL. Echocardiography revealed an ejection fraction of 39% and global wall hypokinesis. Chest X-ray 12 hours after surgery revealed severe interstitial and alveolar oedema (Figure 1D). Pathology revealed a paraganglioma (Figure 2). The patient developed MOF and malignant arrhythmia and ultimately died thirty-six hours later.

**Informed consent**: Informed consent has been obtained from all individuals included in this study.

**Ethical approval**: The research related to human use has been complied with all the relevant national regulations, institutional policies and in accordance the tenets of the Helsinki Declaration, and has been approved by the authors’ institutional review board or equivalent committee.

## 3 Discussion

Intrathoracic extra adrenal extra adrenal paragangliomas are rare tumours of the posterior mediastinum, according to reports over the last decade1. Paragangliomas of the posterior mediastinum are usually functional and affect young people; however, non-functional and even asymptomatic but functional paragangliomas of the posterior mediastinum have been described2. The most common complaints of functional extra adrenal paragangliomas are headache, sweating, tachycardia or palpitations, chest pain, dyspnoea and nausea. The golden standard test for diagnosing of functional pheochromocytoma depends on testing catecholamines and their metabolites in plasma and urine. Blood catecholamine levels more than 2000 pg/ml are considered highly specific for paragangliomas. Iodine-MIBG scintigraphy is a very useful method for locating ectopic pheochromocytomas with high specificity (100%) but low sensitivity (84%)3. Some small hypofunctional tumours can be misdiagnosed due to low isotope uptake. CT and MRI play major roles in detecting extra adrenal paragangliomas and provide the best localization diagnosis. Maybe in our case the chest CT hypervascularized aspect of the mediastinal mass could have suggested the possibility of a pre-operative embolization to reduce the intra-operative release of catecholamines. Nevertheless, asymptomatic paragangliomas that is totally clinically silent is difficult to diagnosis pre-operatively (because there is no cathecholamine secretion outside of tumor, and therefore, they are clinically silent, especially if located in unusual sites, where it can be easily missed or diagnosed and treated as a benign tumour. This is the reason why many extra adrenal paragangliomas are missed until autopsy or surgery. Nevertheless, lethal cases are rare. The causes of death in such cases are often consequences of severe paroxysmal hypertension such as cerebral vascular accidents or acute left ventricular failure caused by massive release of noradrenalin caused by surgery, as in the present case, where the first presentation of a catecholamine-secreting tumour was sudden cardio-respiratory failure and death following the procedure. Few studies report sudden death due to a catecholamine-secreting tumor4. Chiu et al5. reported a case of life-threatening cardiac shock caused by minor abdominal trauma with haemorrhagic pheochromocytoma; the use of ECMO to provide cardiopulmonary life support enabled good recovery. A case of sudden death due to a non-diagnosed pheochromocytoma was provided from Preuß et al6, who reported sudden death in a 49-year-old man due to a pheochromocytoma. L. Andrello7 described a case of sudden death in a 48-year-old woman after a percutaneous alcohol injection into a tumor revealing to be a paraganglioma. Autopsy findings included marked lung congestion and oedema; the myocardium showed widespread fibre fragmentation and many contraction bands, similar to the findings in our case. The blood pressure increased to 210/125 mmHg intraoperatively because a large dose of catecholamine was secreted. When the tumour was completely resected, the blood pressure dropped sharply to 25/15 mmHg. This was a life-threatening and irreversible blood pressure drop that was refractory to fluid and vasopressors. Tolerance of tissue receptors to catecholamines could be the reason. Ultimately, the patient died of multi-organ failure (acute heart failure, circulation failure, hypoxia and metabolic acidosis).

In conclusion, any mass in the posterior mediastinum should be suspected of being a paraganglioma. Asymptomatic paraganglioma can be non-functional but can also be fatal.
